# Healthcare workers’ perspectives on access to sexual and reproductive health services in the public, private and private not-for-profit sectors: insights from Kenya, Tanzania, Uganda and Zambia

**DOI:** 10.1186/s12913-022-08249-y

**Published:** 2022-07-06

**Authors:** Gaby I. Ooms, Janneke van Oirschot, Dorothy Okemo, Tim Reed, Hendrika A. van den Ham, Aukje K. Mantel-Teeuwisse

**Affiliations:** 1 Health Action International, Overtoom 60-2, 1054 HK Amsterdam, The Netherlands; 2 Utrecht WHO Collaborating Centre for Pharmaceutical Policy and Regulation, Division of Pharmacoepidemiology and Clinical Pharmacology, Utrecht Institute for Pharmaceutical Sciences (UIPS), Utrecht University, Utrecht, The Netherlands; 3 Access to Medicines Platform Kenya, Nairobi, Kenya

**Keywords:** Sexual and reproductive health, Sub-Saharan Africa, Health facilities, Healthcare workers, Barriers to access

## Abstract

**Background:**

Access to sexual and reproductive health services remains a challenge for many in Kenya, Tanzania, Uganda and Zambia. Health service delivery in the four countries is decentralised and provided by the public, private and private not-for-profit sectors. When accessing sexual and reproductive health services, clients encounter numerous challenges, which might differ per sector. Healthcare workers have first-hand insight into what impediments to access exist at their health facility. The aim of this study was to identify differences and commonalities in barriers to access to sexual and reproductive health services across the public, private and private not-for-profit sectors.

**Methods:**

A cross-sectional survey was conducted among healthcare workers working in health facilities offering sexual and reproductive health services in Kenya (*n* = 212), Tanzania (*n* = 371), Uganda (*n* = 145) and Zambia (*n* = 243). Data were collected in July 2019. Descriptive statistics were used to describe the data, while binary logistic regression analyses were used to test for significant differences in access barriers and recommendations between sectors.

**Results:**

According to healthcare workers, the most common barrier to accessing sexual and reproductive health services was poor patient knowledge (37.1%). Following, issues with supply of commodities (42.5%) and frequent stockouts (36.0%) were most often raised in the public sector; in the other sectors these were also raised as an issue. Patient costs were a more significant barrier in the private (33.3%) and private not-for-profit sectors (21.1%) compared to the public sector (4.6%), and religious beliefs were a significant barrier in the private not-for-profit sector compared to the public sector (odds ratio = 2.46, 95% confidence interval = 1.69–3.56). In all sectors delays in the delivery of supplies (37.4-63.9%) was given as main stockout cause. Healthcare workers further believed that it was common that clients were reluctant to access sexual and reproductive health services, due to fear of stigmatisation, their lack of knowledge, myths/superstitions, religious beliefs, and fear of side effects. Healthcare workers recommended client education to tackle this.

**Conclusions:**

Demand and supply side barriers were manifold across the public, private and private not-for-profit sectors, with some sector-specific, but mostly cross-cutting barriers. To improve access to sexual and reproductive health services, a multi-pronged approach is needed, targeting client knowledge, the weak supply chain system, high costs in the private and private not-for-profit sectors, and religious beliefs.

**Supplementary Information:**

The online version contains supplementary material available at 10.1186/s12913-022-08249-y.

## Introduction

Sexual and reproductive health and rights (SRHR) encompass “efforts to eliminate preventable maternal and neonatal mortality and morbidity, to ensure quality sexual and reproductive health services, including contraceptive services, and to address sexually transmitted infections (STI) and cervical cancer, violence against women and girls, and sexual and reproductive health needs of adolescents” [1]. Unfortunately, many in Kenya, Tanzania, Uganda and Zambia have poor access to the sexual and reproductive health (SRH) services that address these issues. As a result, their rights are not fulfilled which results in poor SRHR outcomes. Unintended pregnancy rates are high, which range from 105 per 1,000 women aged 15–49 in Tanzania, to 145 per 1,000 women in Uganda, especially when compared to the rate in Europe and Northern America (35 per 1,000 women) [2]. In addition, each year, 340,000 women and 370,000 new-borns in Tanzania do not receive the care they need for major (obstetric) complications, with similar numbers found in Kenya, Uganda and Zambia [2]. Related, the maternal mortality rate remains high in these countries, ranging from 213 per 100,000 live births in Zambia to 524 per 100,000 live births in Tanzania [3]. Further, studies on the prevalence of STIs have shown infection rates to be high, especially among adolescents. In Kenya, two studies investigating the prevalence of chlamydia trachomatis among women found it to be around 11–13%, while a study in Uganda among more than 8,000 adolescents found a 19% self-reported history of STIs [4–6]. Much thus still needs to be done to ensure the SRHRs of people in these countries are fulfilled.

In each country, service delivery is undertaken by three entities: the public sector, the private sector, and the private not-for-profit (PNFP) sector, which for a large part comprise faith-based organisations [7]. In Zambia, the public sector owns 88% of registered facilities, the private sector 13% and the PNFP sector 6% [8]. Ownership in Tanzania is comparable, with the public sector owning 74% of facilities, and the private and PNFP sectors 14% and 13%, respectively [9]. In Kenya and Uganda, ownership between the public and private sector is more evenly distributed, with about 45% public and about 40% private sector ownership [10, 11].

It is known that women and girls encounter numerous challenges in accessing SRH services. On the demand side, barriers include, amongst others, lack of knowledge on SRH, socio-cultural and religious beliefs and practices, poverty, stigmatisation, and healthcare workers’ (HCWs) negative attitudes [12–15]. On the supply side, barriers include unavailability and unaffordability of commodities and services, stockouts, distance to health facilities, staff shortages, and poorly trained HCWs [12–16]. It is, however, unknown how these barriers compare between the three sectors that deliver SRH services. Previous research studied only one sector [17–19], studied multiple sectors but did not stratify results per sector [12], or did not specify which sector(s) were studied [14, 15], which does not allow for comparison across sectors. One study that did measure the availability, affordability and stockouts of sexual and reproductive health commodities (SRHC) across the three sectors found that availability was comparable across sectors, while affordability for specific commodities was only problematic in the private and PNFP sectors [20].

It is essential to know more about how barriers to delivering SRH services vary across sectors. Among others, they have their own supply systems, methods of operation, and offering and pricing of services. Knowing what barriers play out in each of the sectors and how they compare can help to identify the need for and development of sector-specific action plans to address these barriers. The current study addresses this gap. It used a survey design to gather the perspectives of HCWs on the impediments to access to SRH services at their health facility. HCWs have first-hand insight on issues in service provision from their day-to-day work and can provide insights into barriers on both the supply and demand side. The aim of this study was to compare the barriers to access to SRH services across the public, private and private not-for-profit sectors of Kenya, Uganda, Tanzania and Zambia.

## Methods

### Study design and setting

A cross-sectional survey among HCWs in health facilities providing SRH services was conducted in Kenya, Tanzania, Uganda, and Zambia. These countries were selected due to their similar health system structures and comparable performance on SRH indicators [2–6, 8–11].

### Study participants and sampling procedures

HCWs, at the forefront of care delivery, were used as key informants in this study. The HCW needed to be a licensed HCW providing SRH services and had to have worked at the facility for at least one year. The definition of ‘HCW’ included pharmacists, physicians, nurses, and clinical officers.

The sampling strategy used was based on the standardised World Health Organization’s (WHO)/Health Action International’s (HAI) methodology, *Measuring medicine prices, availability, affordability and price components*, in which each country’s main urban region was selected, and in addition five or more other regions were randomly selected for inclusion [21]. This sampling strategy has been shown to be a representative presentation of surveyed countries’ price, availability and affordability situation through a validation study [22]. Regions chosen included ten counties in Kenya, twelve districts in Tanzania, six regions in Uganda, and ten provinces in Zambia. In each survey region, at least 24 facilities, located in both urban and rural areas, were randomly selected from the public, private and PNFP sectors. Facilities where HCWs were working had to be within three hours travel from the main public provincial health facility. In total, the target sample size consisted of 912 HCWs in Kenya (*n* = 240), Tanzania (*n* = 288), Uganda (*n* = 144), and Zambia (*n* = 240). Urban was defined based on the definition used by the countries’ national bureaus of statistics [23]. The healthcare levels included in the study ranged from the health post/dispensary level to regional and national (referral) hospitals. In each facility, one HCW was asked to participate in the survey.

### Data collection and tool

The survey collected information about the HCWs’ perceptions on the SRH services offered at their facility, key challenges to accessing SRH services, perspectives on SRHC stockouts, perspectives on clients’ potential reluctance to access SRH services, and recommendations to improve access. The survey was developed in collaboration with in-country civil society experts, and consisted of seven open-ended and three close-ended questions (see Supplementary file 1). The survey was pilot-tested in 2018 in all four countries, after which it was refined and one question was added based on feedback from in-country experts. Refinement of the survey occurred in phrasing of the questions, and specification within the questions between supply- and demand-side barriers. Data were collected using a mobile data collection application in July 2019. In each country, local consultants specialised in this type of research undertook the data collection. They were trained during a two-day workshop by the authors (GO, DO), after which they piloted the survey during a field test. The local consultants worked in pairs and were supervised by an in-country lead. The survey took on average twenty minutes to complete.

### Data management and analysis

Data were regularly uploaded to the server and downloaded into Microsoft Excel after completion of the data collection. Data were double-checked by the researchers, responses were verified with the data collectors when questions about their meaning arose, and open-ended questions were categorised. Thereafter, data were imported into Stata version 17 for analysis. Simple descriptive statistics were used to describe the data, while binary logistic regression analyses were used to test for significant differences in access barriers and recommendations between sectors. In the analyses we controlled for country, location (urban vs. rural), and level of care of the health facility. Odds ratios (ORs) and 95% confidence intervals (95% CIs) were reported to assess if some answers were more (or less) likely to be mentioned by HCWs in the private sector and PNFP sector compared to the public sector. A significance cut-off value of 0.05 was used.

### Ethical considerations

Ethical approval for the study was obtained from the Amref Ethics and Scientific Review Committee (P394-2017) and National Commission for Science & Technology (NACOSTI/P/19/36,482/31,905) in Kenya, the National Institute for Medical Research in Tanzania (NIMR/HQ/R.8a/Vol. IX/2797), the Makerere University School of Health Sciences in Uganda (2018-017), and ERES Converge in Zambia (2018-Apr-010). Further, permission was granted by letter by the respective county/district Directors of Health and Ministries of Health. Participants were provided with an information sheet, and their informed consent was obtained orally before the survey was undertaken. No identifying information was collected about the participants, and all data was stored on password-protected computers.

## Results

In total, 971 HCWs participated from Kenya (*n* = 212), Tanzania (*n* = 371), Uganda (*n* = 145) and Zambia (*n* = 243) (see Table 1). More than half of HCWs worked in the public sector, 25.9% worked in the private sector, and 19.5% in the PNFP sector. HCWs believed that family planning services experienced the most access challenges (41.2%), followed by maternal health (27.7%) and STI management (22.4%) services. Only 8.7% of HCWs indicated child health services to experience most access challenges of the SRH services.

**Table 1:** Characteristics of study participants

	N	%
Country		
Kenya	212	21.8
Tanzania	371	28.2
Uganda	145	14.9
Zambia	243	25.0
Sector		
Public	531	54.7
Private	251	25.9
PNFP	189	19.5
Area		
Urban	530	54.6
Rural	441	45.4
Level^a^		
I	416	42.8
II	190	19.6
III	235	24.2
IV	79	8.1
V	51	5.3

^a^Health facility levels in Kenya: (I) Dispensary/clinic, pharmacy; (II) Health centre; (III) Primary hospital; (IV) Secondary care hospital; (V) Teaching/national hospital. In Tanzania: : (I) Dispensary/clinic, pharmacy; (II) Health centre; (III) Council hospital; (IV) Regional referral hospital; (V) Zonal/national hospital. In Uganda: (I) Dispensary/clinic, pharmacy; (II) Health centre II; (III) Health centre III; (IV) Health centre IV; (V) (Regional referral) hospital. In Zambia: (I) Dispensary/clinic, pharmacy; (II) Health post; (III) Health centre; (IV) District hospital; (V) General hospital and above

### HCWs’ perspectives on access to SRH per sector

When HCWs were asked about the key challenges to accessing SRHC, the most commonly mentioned barrier in the public sector was issues with the supply to the health facility (42.5%). In the private sector patients’ lack of knowledge (37.0%) was most often mentioned, which was also commonly mentioned in the other sectors (see Table 2). In the PNFP sector the barrier most cited was religious or cultural beliefs on both the supply- and demand side (44.9%); HCWs in this sector had higher odds (OR = 2.46, 95% CI = 1.69–3.56) of mentioning this barrier than their counterparts in the public sector. In the private and PNFP sectors, HCWs were less likely to mention issues with the supply to the health facility, frequent stockouts at the health facility, and staff shortages than HCWs in the public sector. In the private sector, HCWs were also less likely to indicate staff training on SRH as a key challenge to accessing SRHC than those in the public sector (9.8% vs. 19.3%, OR = 0.49, 95% CI = 0.28–0.83), while in the PNFP sector HCWs were less likely to mention stockouts at the central level as a barrier than HCWs in the public sector (4.3% vs. 13.6%, OR = 0.35, 95% CI = 0.16–0.75). Both the HCWs in the private (33.3%, OR = 6.83, 95% CI = 3.98–11.70) and PNFP sectors (21.1%, OR = 4.58, 95% CI = 2.61–8.03) were more likely to mention patient costs as barrier than HCWs from the public sector (4.6%).

**Table 2:** HCW perspectives on access to SRH barriers and recommendations for improvement, per sector

	Overall N (%)	Public N (%)	Private N (%)	OR (95% CI)^a^	PNFP N (%)	OR (95% CI)^a^
Key challenges to accessing SRHC						
Patient lack of knowledge on SRH	354 (37.1)	203 (38.8)	91 (37.0)	0.99 (0.69–1.42)	60 (32.4)	0.75 (0.52–1.09)
Issues with supply to HF	320 (33.5)	222 (42.5)	56 (22.8)	0.40*** (0.27–0.59)	42 (22.7)	0.44*** (0.29–0.65)
Frequent stockouts at HF	282 (29.6)	188 (36.0)	49 (19.9)	0.47*** (0.31–0.72)	45 (24.3)	0.57** (0.38–0.85)
Religious/cultural beliefs	272 (28.5)	142 (27.2)	47 (19.1)	0.75 (0.50–1.15)	83 (44.9)	2.46*** (1.69–3.56)
Stigma	207 (21.7)	113 (21.6)	56 (22.8)	0.97 (0.63–1.48)	38 (20.5)	0.75 (0.49–1.16)
Staff shortages	182 (19.1)	144 (27.5)	18 (7.3)	0.26*** (0.15–0.46)	20 (10.8)	0.34*** (0.21–0.57)
Staff training on SRH services	148 (15.5)	101 (19.3)	24 (9.8)	0.49** (0.28–0.83)	23 (12.4)	0.61 (0.37–1.01)
Patient costs	145 (15.2)	24 (4.6)	82 (33.3)	6.83*** (3.98–11.70)	39 (21.1)	4.58*** (2.61–8.03)
No demand	102 (10.7)	40 (7.7)	45 (18.3)	1.30 (0.74–2.28)	17 (9.2)	1.02 (0.55–1.91)
Frequent stockouts at central level	102 (10.7)	71 (13.6)	23 (9.4)	0.70 (0.39–1.25)	8 (4.3)	0.35** (0.16–0.75)
SRHC stockout causes						
Delay in supply delivery	471 (54.1)	320 (63.9)	83 (37.4)	0.36 (0.24–0.54)***	68 (46.0)	0.52** (0.34–0.77)
What is ordered is not what HF received	295 (33.9)	226 (45.1)	37 (16.7)	0.31 (0.20–0.49)***	32 (21.6)	0.35*** (0.22–0.54)
Problems with stock at medical stores	264 (30.3)	170 (33.9)	61 (27.5)	0.94 (0.62–1.41)	33 (22.3)	0.61* (0.39–0.95)
Demand higher than availability	185 (21.2)	120 (24.0)	37 (16.7)	0.49** (0.31–0.81)	28 (18.9)	0.76 (0.47–1.23)
Affordability for HF	138 (15.8)	33 (6.6)	67 (30.2)	5.59*** (3.27–9.53)	38 (25.7)	4.82*** (2.79–8.34)
Poor stock management at HF	128 (14.7)	63 (12.6)	34 (15.3)	1.37 (0.81–2.32)	31 (21.0)	1.84* (1.11–3.04)
Lack of storage space at HF	80 (9.2)	58 (11.6)	14 (6.3)	0.54 (0.28–1.08)	8 (5.4)	0.48 (0.21–1.07)
Problems with medicine transport to HF	71 (8.2)	51 (10.2)	10 (4.5)	0.49 (0.22–1.08)	10 (6.8)	0.67 (0.32–1.39)
Recommendations for improvement – supply side						
Improve supply chain	523 (55.6)	346 (66.4)	104 (43.2)	0.40*** (0.27–0.57)	73 (41.0)	0.38*** (0.27–0.56)
Timely supply of SRHC	430 (45.7)	274 (52.6)	84 (34.9)	0.48*** (0.33–0.70)	72 (40.5)	0.61** (0.42–0.87)
Prevent stockouts of SRHC at HF	326 (34.7)	192 (36.9)	80 (33.2)	1.04 (0.71–1.50)	54 (30.3)	0.75 (0.51–1.10)
Ensure sufficient stock available at HF	275 (28.7)	180 (34.2)	56 (22.6)	0.65* (0.44–0.97)	39 (21.2)	0.57** (0.38–0.85)
Supply SRHC that were ordered	247 (26.3)	179 (34.4)	46 (19.1)	0.56** (0.37–0.86)	22 (12.4)	0.28*** (0.17–0.46)
(Continued) staff training	216 (23.0)	140 (26.9)	42 (17.4)	0.63* (0.41–0.97)	34 (19.1)	0.66 (0.43–1.03)
Increase staff	203 (21.6)	143 (27.5)	30 (12.5)	0.51** (0.32–0.82)	30 (16.9)	0.57* (0.36–0.90)
Increase budget for SRHC	176 (18.7)	112 (21.5)	33 (13.7)	0.50** (0.30–0.81)	31 (17.4)	0.76 (0.48–1.20)
Provide greater choice of SRHC	147 (15.6)	71 (13.6)	49 (20.3)	1.60* (1.00-2.55)	27 (15.2)	1.05 (0.63–1.73)
Recommendations for improvement – demand side						
Client and community education	778 (81.1)	437 (82.9)	194 (78.2)	0.77 (0.50–1.20)	147 (79.9)	0.89 (0.57–1.39)
Increase male partner involvement	357 (37.2)	222 (42.1)	82 (33.1)	0.82 (0.57–1.18)	53 (28.8)	0.57** (0.39–0.83)
Offer/improve SRH outreach services	280 (29.2)	164 (31.1)	62 (25.0)	0.77 (0.52–1.14)	54 (29.4)	0.86 (0.58–1.26)
Increase choice of contraceptives	222 (23.2)	129 (24.5)	59 (23.8)	0.76 (0.50–1.16)	34 (18.5)	0.76 (0.49–1.18)
Professionalise HCW-patient relationship	173 (18.0)	102 (19.4)	49 (19.8)	0.88 (0.56–1.36)	22 (12.0)	0.43** (0.26–0.73)
Reduce costs for clients	202 (21.0)	38 (7.2)	113 (45.2)	7.60*** (4.79–12.04)	51 (27.7)	4.10*** (2.53–6.63)
HF at times unable to provide client with SRHC and services						
Yes	359 (37.0)	155 (29.2)	123 (49.0)	1.57* (1.09–2.26)	81 (42.9)	1.47* (1.02–2.12)
Reasons why unable to provide client with SRHC and services						
SRHC was stocked out	131 (37.3)	84 (56.4)	35 (28.2)	0.30*** (0.16–0.56)	12 (15.4)	0.11*** (0.07–0.28)
HF does not offer FP services	65 (18.6)	13 (8.8)	24 (19.5)	1.88 (0.82–4.30)	28 (35.9)	6.38*** (2.97–13.72)
Client unable to pay for service	60 (17.2)	4 (2.7)	44 (35.8)	15.13*** (4.85–47.18)	12 (15.4)	6.88** (2.08–22.70)
Client was too young	58 (16.6)	19 (12.8)	26 (21.1)	1.72 (0.78–3.83)	13 (16.7)	1.15 (0.51–2.60)
Service not culturally or religiously acceptable	56 (16.1)	13 (8.7)	5 (4.1)	0.42 (0.13–1.37)	38 (49.4)	12.65*** (5.75–27.81)
Service would not benefit client	25 (7.2)	11 (7.4)	9 (7.3)	1.26 (0.42–3.81)	5 (6.4)	0.60 (0.19–1.90)
Lack of HCW knowledge	23 (6.6)	16 (10.7)	5 (4.0)	0.53 (0.16–1.74)	2 (2.6)	0.22* (0.05–0.99)
Client was unmarried	17 (4.9)	6 (4.1)	4 (3.3)	0.59 (0.13–2.64)	7 (9.0)	1.63 (0.49–5.45)

*CI* confidence interval, FP family planning, HCW healthcare worker, HF health facility, OR odds ratio, SRH  sexual and reproductive health, SRHC sexual and reproductive health commodities

*p < 0.05, **p < 0.01, ***p < 0.001

^a^The model was corrected for country, location, and level of care of the health facility

When HCWs were asked about the causes of SRHC stockouts at their facilities, in all sectors they most commonly said that it was due to delays in the delivery of the SRHC (37.4-63.9%). In the public sector, another commonly mentioned cause of SRHC stockouts was a difference between supplies ordered by the facility, and those received (45.1%). Both of these reasons were less likely to be mentioned as a cause of stockouts in the private and PNFP sector. HCWs in these two sectors did have a 5.59 (95% CI = 3.27–9.53) and 4.82 (95% CI = 2.79–8.34) higher odds, respectively, of giving poor affordability of SRHC as a reason for stockouts than in the public sector.

HCWs also shared what they believed could be done, on both the supply- and demand side, to improve access to SRHC. On the supply side, the most often shared recommendation was the general recommendation to improve the supply chain (41.0-66.4%). Nevertheless, HCWs in the private (OR = 0.40, 95% CI = 0.27–0.57) and PNFP (OR = 0.38, 95% CI = 0.27–0.56) sectors were less likely to mention this recommendation than HCWs in the public sector. Ensuring the timely supply of SRHC and preventing stockouts of SRHC at the facility were also commonly provided recommendations across the three sectors. Public sector HCWs also often recommended increasing number of staff offering SRH services (27.5%) and increasing staff training on SRH service provision (26.9%).

To improve the demand for commodities, more than 80% of HCWs across the sectors saw a need for community education on SRH. Offering or improving outreach services and increasing male partner involvement were also commonly recommended across the sectors. Nevertheless, PNFP sector HCWs were less likely to recommend increasing male partner involvement than public sector HCWs (42.1% vs. 28.8%, OR = 0.57, 95% CI = 0.39–0.83). In the private and PNFP sectors, HCWs were more likely to recommend reducing costs for clients than their counterparts in the public sector (OR = 7.60, 95% CI = 4.79–12.04 and OR = 4.10, 95% CI = 2.53–6.63, respectively).

HCWs were also asked if they were at times unable to provide clients with SRHC and SRH services; 29.2% of HCWs in the public sector indicated this was the case, with HCWs in the private sector (49.0%) and PNFP sector (42.9%) being significantly more likely to state they experienced this issue. The most commonly provided reason for this in the public sector was that the SRHC was out of stock (56.4%), which was a less likely reason given in the private (28.2%, OR = 0.30, 95% CI = 0.16–0.56) and PNFP (15.4%, OR = 0.14, 95% CI = 0.07–0.28) sectors. In the private sector, the most indicated reason was that clients were unable to pay for the service (35.8%).The most common reasons given in the PNFP sector were because the service was not culturally or religiously acceptable (49.4%) and because the health facility did not offer family planning services (35.9%).

Further, 39.3% of HCWs thought that clients were reluctant to access SRHC (see Table 3). The most commonly provided reasons for clients’ reluctance were fear of stigmatisation (63.0%), patients’ lack of knowledge (50.0%), myths or superstitions (44.7%), religious beliefs (39.2%) and fear of side effects (38.6%). HCWs from the PNFP sector were less likely (OR = 0.43, 95% CI = 0.19–0.97) than public sector HCWs to believe low support from male partners was a reason for client reluctance. Conversely, they were more likely (OR = 2.46, 95% CI = 1.05–5.73) to believe poverty and costs played a role in their reluctance.

To tackle clients’ reluctance, almost all HCWs (97.4%) recommended expanding client education. Other commonly mentioned recommendations included creating youth-friendly health corners (35.8%) and involving partners in the SRH care (28.9%). The youth-friendly health corners were less likely to be recommended by HCWs from the private and PNFP sectors than by those from the public sector, while involving partners was also less likely to be recommended by PNFP sector HCWs compared to public sector HCWs. Staff training was also less likely to be recommended by HCWs from the private (OR = 0.46, 95% CI = 0.21–0.99) and PNFP (OR = 0.43, 95% CI = 0.20–0.95) sectors than by those in the public sector. These HCWs were more likely than public sector HCWs to recommend reducing costs for patients to tackle their reluctance. In the PNFP sector, HCWs were also more likely (OR = 3.19, 95% CI = 1.18–8.60) to recommend providing free family planning services than their counterparts in the public sector.

 The presented adjustments in the models for country, location, and level of care of the facility did not substantially change the results compared to the crude results (see Supplementary file 2). The barriers and recommendations shared by the HCWs were comparable across the four countries (see Supplementary file 3).

**Table 3:** HCW perspectives on client reluctance to access SRH services, per sector

	Overall N (%)	Public N (%)	Private N (%)	OR (95% CI)^a^	PNFP N (%)	OR (95% CI)^a^
Clients reluctant to access SRH services						
Yes	381 (39.3)	195 (36.7)	108 (43.0)	1.03 (0.72–1.49)	78 (41.5)	0.92 (0.64–1.31)
Reasons for reluctance to access SRH services						
Fear of stigmatisation	238 (63.0)	115 (59.6)	70 (65.4)	0.69 (0.36–1.32)	53 (68.0)	0.83 (0.44–1.58)
Patient lack of knowledge	189 (50.0)	100 (51.8)	57 (53.3)	0.96 (0.53–1.73)	32 (41.0)	0.64 (0.36–1.15)
Myths or superstitions	169 (44.7)	95 (49.2)	43 (40.2)	0.86 (0.48–1.56)	31 (39.7)	0.83 (0.47–1.48)
Religious beliefs	148 (39.2)	84 (43.5)	33 (30.8)	0.89 (0.47–1.67)	31 (39.7)	1.40 (0.76–2.59)
Fear of side effects	146 (38.6)	71 (36.8)	46 (43.0)	1.45 (0.78–2.68)	29 (37.2)	0.88 (0.48–1.62)
Low support - male partner	78 (20.6)	49 (25.4)	20 (18.7)	0.64 (0.31–1.33)	9 (11.5)	0.43* (0.19–0.97)
Poverty/costs	48 (12.7)	13 (6.7)	20 (18.7)	2.14 (0.85–5.38)	15 (19.2)	2.46 (1.05–5.73)*
Frequent stockouts at HF	32 (8.5)	23 (11.9)	4 (3.7)	0.31 (0.08–1.19)	5 (6.4)	0.58 (0.20–1.73)
Distance to HF	28 (7.4)	18 (9.3)	5 (4.7)	1.24 (0.34–4.50)	5 (6.4)	0.68 (0.21–2.15)
Low support - female partner	21 (5.6)	10 (5.2)	7 (6.5)	0.99 (0.28–3.52)	4 (5.1)	1.00 (0.27–3.72)
Recommendations to tackle client reluctance						
Expand client education	367 (97.4)	189 (97.4)	101 (97.1)	0.78 (0.11–5.68)	77 (97.5)	1.39 (0.19–10.42)
Create youth-friendly health corners	135 (35.8)	76 (39.2)	35 (33.7)	0.43* (0.21–0.84)	24 (30.4)	0.42* (0.22–0.82)
Involve partners	109 (28.9)	67 (34.5)	26 (25.0)	0.56 (0.29–1.08)	16 (20.3)	0.46* (0.24–0.91)
Staff training	75 (19.9)	45 (23.2)	19 (18.3)	0.46* (0.21–0.99)	11 (13.9)	0.43* (0.20–0.95)
Improve HCW-patient relationship	63 (16.7)	33 (17.0)	17 (16.4)	0.84 (0.39–1.84)	13 (16.5)	0.77 (0.36–1.65)
Improve stock availability	57 (15.1)	34 (17.5)	15 (14.4)	0.56 (0.23–1.33)	8 (10.1)	0.48 (0.20–1.15)
Empower people economically	51 (13.5)	18 (9.3)	22 (21.2)	1.70 (0.69–4.18)	11 (13.9)	1.24 (0.52–2.96)
Reduce costs for patients	36 (9.6)	5 (2.6)	23 (22.1)	6.97** (2.20-22.07)	8 (10.1)	3.47* (1.04–11.56)
Provide free FP services	32 (8.5)	9 (4.6)	11 (10.6)	1.95 (0.66–5.77)	12 (15.2)	3.19* (1.18–8.60)

*CI* confidence interval, *FP *family planning, *HCW *healthcare worker, *HF *health facility, *OR *odds ratio, *SRH *sexual and reproductive health

*p < 0.05, **p < 0.01, ***p < 0.001

^a^The model was corrected for country, location, and level of care of the health facility

## Discussion

This study looked at what barriers to accessing SRH services exist at both the supply- and demand side in the public, private and PNFP sectors and what ought to be done to improve the situation, from the perspective of HCWs. It found that some significant differences existed in perspectives of HCWs across the different sectors, even though in general many barriers were cross-cutting. One of the most commonly raised barriers to accessing SRH services was patient lack of knowledge. Issues with supply of commodities and frequent stockouts were often raised in the public sector. Patient costs were a significant barrier in the private and PNFP sectors, and religious and cultural beliefs were commonly mentioned in the PNFP sector. HCWs in all sectors mentioned delay in delivery of supplies as main reason for stockouts, with affordability of commodities being a significant problem in the private and PNFP sectors. Further, HCWs believed that clients were often reluctant to access SRH services, caused by fear of stigmatisation, their lack of knowledge, myths and superstitions, religious beliefs, and fear of side effects. Main recommendations to improve access were similar across the sectors and in line with the raised barriers.

Patient lack of knowledge about SRH and SRH services, raised as a main challenge by HCWs across the sectors, is an often-raised barrier to accessing SRH services [24–27]. Related to this, HCWs believed that clients’ reluctance to access SRH services was caused for a large part by their lack of knowledge, as well as myths or superstitions, and fear of side effects. Again, this has been well-documented elsewhere, and has been perceived by both HCWs and clients themselves as barriers [14, 25, 27–29]. Thus, more should be done to improve clients’ knowledge about SRH services and commodities, including on offered services, on how to properly use certain commodities (e.g. condoms), and on true side effects of commodities (e.g. the birth control pill). This because many misunderstandings persist, including that contraceptives cause infertility [14, 28, 29]. However, research has shown that only tackling client knowledge may only have a limited effect on health-seeking behaviour [24, 25]. A multi-pronged approach is thus needed, tackling the other factors which also influence access to SRH services.

For instance, religious and cultural beliefs were also seen as one of the key challenges to accessing SRH services. Especially in the PNFP sector, which in these countries constitutes for the most part faith-based facilities, it seemed to negatively impact access. HCWs in this sector who indicated they were at times unable to provide clients with SRH services gave as most common reasons that the service was not culturally or religiously acceptable and that the health facility did not offer family planning services. These arguments were both much less relevant across the other sectors.

Research has shown that adolescents saw unsupportive attitudes from HCWs as a major barrier to access to SRH services. In contrast, the HCWs themselves did not think their attitudes interfered with the use of services among adolescents [26]. In other studies, however, HCWs did recognise that HCWs’ negative attitudes impacted access [19, 30]. Previous research has shown that some HCWs might be reluctant to provide family planning services because they believe the use of any type of contraceptive is inappropriate, especially to adolescents or unmarried women and girls [14, 18, 19]. Our study found that HCWs who work at PNFP sector facilities acknowledge that religious beliefs form a barrier to access to SRH services. Many Catholic health facilities in the four countries also do not provide contraceptives, with the exception of condoms, which forms a significant issue for those dependent on these facilities for their healthcare services [31, 32]. HCWs, especially those in PNFP sector facilities, are an important group to target for continuous education. Improvements in their knowledge and attitudes will improve access to services [33]. Secondly, engaging them in campaigns with civil society and communities to fulfil a more activist role can be a powerful tool to improve access [34].

Next to knowledge and attitudinal barriers, this study also highlighted the high costs of care to patients in the private and PNFP sectors. This finding is not surprising, as out-of-pocket health expenditure in the countries ranges from 10% of all health expenditure in Zambia, to 38% of all health expenditure in Uganda [35]. In sub-Saharan Africa, many countries are focusing on attaining universal health coverage (UHC). They often establish public-private partnerships (PPPs), through which the government collaborates with the private sector to provide health services [36]. As part of these PPPs, countries are implementing prepayment health financing schemes such as social insurance or national health insurance (NHI). Members of such schemes pay a fee which allows them to access care at private facilities for ‘free’, with private facilities reimbursed for the care provided [37]. However, rollout of NHI schemes differs across the four countries. About 15% and 30% of Kenya’s and Tanzania’s population is covered by such a scheme, while in Zambia, as of October 2021, only 191 of 1956 registered health facilities had been accredited. Uganda has no NHI in existence yet [8, 38–41].

PPPs and NHI can be useful tools to reduce costs for clients and improve access to medicines when it is functioning well and has a high population coverage [42–44].However, at the moment many bottlenecks exist in the two study countries where NHI has been implemented for a longer time that limit its potential. Premiums paid by the insured are unaffordable to parts of the population, stockouts or lack of commodities at facilities force clients to buy out-of-pocket at non-accredited facilities, shortages of HCWs affect quality of services, a pro-urban distribution of health facilities results in clients needing to travel long distances to accredited facilities in rural areas, and delays in provider reimbursement by the NHI scheme result in co-payments by clients, denial or limiting of services, and long waiting times [39, 40, 45, 46]. To fulfil its potential, governments ought to focus on tackling these bottlenecks.

Logistical problems were also raised by the HCWs as causing significant challenges. These included issues with supply to the facility as well as stockouts, which were said to be caused by delays in deliveries, incorrect orders and deliveries, and problems with the stock at the medical stores. Problems with stockouts have also been identified previously in the four countries [14, 18, 20, 47]. Strengthening the supply chain systems should be one of the main priorities of the countries’ governments. Stockouts can be prevented, or at least minimised, with a well-functioning logistic management information system, staff trained in supply chain management, and sufficient budget allocations to commodity procurement [48].

It is important to note that not only barriers at the provider or supply chain level influence commodity availability and stockouts; they are also influenced by global forces. For instance, sufficient budget allocations to commodity procurement are dependent on the health budget available. These budgets are still dependent on donor funding, making them vulnerable to the whims of donors, and challenging sustainable programme implementation [49–52]. This is especially the case as over the past years, the countries have seen a decrease in this type of funding [49–52]. In Kenya, for example, donor funding made up 33% of the health budget in financial year 2017/18, which decreased to 16% in financial year 2019/20 [53]. Even though the government has increased their own spending on the health budget, it has been inadequate to offset the decrease in donor aid [53]. Further, the global gag rule re-instated and expanded during President Trump’s presidency had far-reaching consequences on access to SRH services far beyond abortion care. In Uganda, for instance, organisations that had lost funding due to the global gag rule were forced to scale down or close down community sensitisation programmes on family planning, outreach services focusing on long-term contraceptives, and health facility collaborations on family planning with community health workers [54]. Another organisation had to shut one of their health facilities due to the lost funding [54]. Last, preferences of international development organisations and donors also impact the availability of commodities. The female condom, for example, invented in 1984, has for decades been met with scepticism and neglect by international development organisations and donors. They referenced a lack of user demand and high prices, resulting in lack of rollout at the national level and subsequent low availability [55]. To offset the impact of global forces and decrease the dependency on donor aid, and ensure sustainable and improved access to SRH services, the governments ought to increasingly and continuously invest in their health systems.

## Strengths and limitations

This study provides quantitative insights into commonalities and differences of the barriers to accessing SRH services across public, private and PNFP sector health facilities in four sub-Saharan African countries. This type of study was selected as it is a proven manner to investigate beliefs and opinions of specific target groups within a limited amount of time, with high representativeness. Although these types of surveys may be prone to socially acceptable answers, we have no indication that this was the case in our study when looking at the results. Further, data collectors were experienced in conducting this type of research and were trained on how to make participants feel safe and comfortable, how to ask questions in an open-ended manner, and how to guarantee the participants’ privacy. A limitation is that we used the experiences of HCWs providing SRH services to identify barriers on both the supply- and demand side. However, they do not have full insights into the barriers as experienced by those seeking SRH services. Therefore, demand side barriers provided here should be considered in that light and not as a complete picture of all barriers clients might experience when accessing SRH services. It is also possible that HCWs might not have been as reflective about their health facilities or colleagues’ shortcomings as clients might have been. Further, logistic regressions were performed to correct for influences of variables such as country, location of health facility and level of health facility, with relatively wide 95% CIs. Less value should therefore be given to the exact ORs and focus should instead be put on the directions of the found associations.

## Conclusions

This study showed that HCWs experienced both demand and supply side barriers across the public, private and PNFP sectors, with some sector-specific, but mostly cross-cutting barriers. To improve access to SRH services across the sectors in the four countries, a multi-pronged approach is needed, targeting these barriers on both the supply- and demand side. Efforts should focus on improving knowledge through client education, HCW sensitisation and education regarding unhelpful religious and cultural beliefs, improving supply chain systems through strengthening logistic management information systems, training staff in supply chain management, and allocating sufficient budget to commodity procurement. Last, unaffordability in the private and PNFP sectors can be tackled through a well-functioning NHI scheme.

## Supplementary Information

**Additional file 1.** HCW survey. 

**Additional file 2.** HCWs perspectives on access to SRH barriers and recommendations for improvement, per sector. Crude and adjusted models.

**Additional file 3.**  HCWs perspectives on access to SRH barriers and recommendations for improvement, per country. Numbers represent percentage of HCWs that mentioned this barrier or recommendation.

## Data Availability

All data generated or analysed during this study are included in this published article and its supplementary information files.

## Abbreviations

95% CI: 95% confidence interval
FP: Family planning
HAI: Health Action International
HCW: Healthcare worker
HF: Health facility
iCHF: Improved community health fund
NHI: National health insurance
NHIF: National health insurance fund
OR: Odds ratio
PNFP: Private not-for-profit
SRH: Sexual and reproductive health
SRHC: Sexual and reproductive health commodities
STI: Sexually transmitted infection
UHC: Universal health coverage
WHO: World Health Organization
