# Supplementary material for: Healthcare workers’ perspectives on access to sexual and reproductive health services in the public, private and private not-for-profit sectors: insights from Kenya, Tanzania, Uganda and Zambia
Source: BMC Health Serv Res. 2022 Jul 6;22:873. doi: 10.1186/s12913-022-08249-y (PMC9261038; doi:10.1186/s12913-022-08249-y)
Supplement: Supplementary file 1 — Additional file 1. HCW survey. [file 12913_2022_8249_MOESM1_ESM.docx]

**Supplementary File 1. HCW survey.**

1. **In your opinion, which of the following categories of Sexual and Reproductive Health (SRH), and Mother and Child Health services (MCH) faces the most challenges related to access to commodities?**

(Choose only one option)

Family Planning Maternal Health STI Management Child Health

1. **Based on the first question, what do you think are the key challenges related to accessing these essential SRH and MCH commodities?**

Do not read. Listen to their answer and choose all that apply. If other answer options are given than those below, choose 'other' and note it on the next page.

There is no demand for the commodities

Issues or delays with supply of the commodities to the facility

Frequent stock-outs at facility level

Frequent stock-outs at central level

Shortage of staff

Training of staff

Costs of medicines to patients

Lack of knowledge of patients about SRH services/commodities available

Religious or cultural beliefs

Perceived stigma associated with accessing the commodities

Health professionals' reluctance to provide certain client groups with commodities or services (because of age, marital status, religion etc.)

Drugs are not supplied as per the supply cycle

Other (specify on next page)

**2a. Other:**__________________________________________________________________

1. **In your opinion, what are the causes of stock-outs of SRH and MCH commodities at your facility?**

Do not read. Listen to their answer and choose all that apply. If other answer options are given than those below, choose 'other' and note it on the next page.

What is ordered is not what the facility received

Delay in supplies being delivered

Poor stock management at the facility

Lack of storage space at the facility

Problems with the transport of the medicines to the facility

Problems with the stock at the central/ distribution level

Affordability of the medicines for the facility

Demand is higher than the supplied medicines

Other (specify on next page)

**3a. Other:**__________________________________________________________________

1. **What can be done to improve access to SRH and MCH commodities and services on the supply side (e.g. healthcare workers, health facilities, suppliers, the health system and the government)?**

Do not read. Listen to their answer and choose all that apply. If other answer options are given than those below, choose 'other' and note it on the next page.

Improve supply chain

Timely supply of the commodities

Supply commodities that have been ordered

Ensure availability of commodities at the facility

Improve stock management (ordering and reporting) at the facility

Increase staff

(Continued) staff training

Reduce costs

Provide greater choice of SRHC

Improve infrastructure

Increase budget for SRH

More supervision from central level

Other (specify on next page)

**4a. Other:**__________________________________________________________________

1. **What can be done to improve access to SRH and MCH commodities and services on the demand side (e.g. the client and community)?**

Do not read. Listen to their answer and choose all that apply. If other answer options are given than those below, choose 'other' and note it on the next page.

Client and community education

Reduce costs

Increase male partner involvement

Professional healthcare worker – patient relationship

Offer/improve outreach SRH services

Increase choice of contraceptives

Ensure enough stocks are available at the facility

Other (specify on next page)

**5a. Other:**__________________________________________________________________

1. **Do you think clients are reluctant to access sexual and reproductive health commodities?**

Yes No

1. **If you answered ‘Yes’, please explain why you think clients are reluctant to access/request SRHC.**

Do not read. Listen to their answer and choose all that apply. If other answer options are given than those below, choose 'other' and note it on the next page.

Fear for stigmatization from family members or the community

Myths or superstition

Religious beliefs

Fear of side effects

Low support from male partners

Low support from female partners

Patient lack of knowledge

Poverty/ costs

Distance to clinic

Frequent stock-outs at the facility

Unprofessional healthcare workers

Other (specify on next page)

**7a. Other:**__________________________________________________________________

1. **How could this reluctance be tackled?**

Do not read. Listen to their answer and choose all that apply. If other answer options are given than those below, choose 'other' and note it on the next page.

Expand client education for ALL (male and female)

Empower people economically

Create youth/adolescent friendly health corners

Involve partners in SRH services

Improve stock availability

Improve/create professional healthcare worker – patient relationships

Training of staff to improve quality of services

Provide free family planning services

Reduce costs for patients

Other (specify on next page)

**8a. Other:**__________________________________________________________________

1. **Are there ever clients that come to your health facility whom you cannot provide with the services or commodities they request?**

Yes No

1. **If yes, please specify why you cannot provide these clients with sexual and reproductive health services or commodities.**

Do not read. Listen to their answer and choose all that apply. If other answer options are given than those below, choose 'other' and note it on the next page.

The client was too young

The client was unmarried

The client was unable to pay for the services

The client did not have health insurance

We do not offer family planning services

I do not believe that the service/commodity would benefit the client

The service requested by the client is not culturally or religiously acceptable

The SRH commodity was stocked out

Lack of knowledge about the service by the HCW

Other (specify on next page)

**10a. Other:**__________________________________________________________________
