# Supplementary material for: Healthcare workers’ perspectives on access to sexual and reproductive health services in the public, private and private not-for-profit sectors: insights from Kenya, Tanzania, Uganda and Zambia
Source: BMC Health Serv Res. 2022 Jul 6;22:873. doi: 10.1186/s12913-022-08249-y (PMC9261038; doi:10.1186/s12913-022-08249-y)
Supplement: Supplementary file 2 — Additional file 2. HCWs perspectives on access to SRH barriers and recommendations for improvement, per sector. Crude and adjusted models. [file 12913_2022_8249_MOESM2_ESM.docx]

**Supplementary File 2.** **HCWs perspectives on access to SRH barriers and recommendations for improvement, per sector.** **Crude and adjusted models.**

|  | | **Overall**  **N (%)** | | **Public**  **N (%)** | | **Private**  **N (%)** | | **OR (95% CI)** | | | | **PNFP**  **N (%)** | | **OR (95% CI)** | | | |
| --- | --- | --- | --- | --- | --- | --- | --- | --- | --- | --- | --- | --- | --- | --- | --- | --- | --- |
|  | |  | |  | |  | | Model 1 | | Model 2^a^ | |  | | Model 1 | | Model 2 ^a^ | |
| Key challenges to accessing SRHC | |  | |  | |  | |  | |  | |  | |  | |  | |
| Patient lack of knowledge on SRH | | 354 (37.1) | | 203 (38.8) | | 91 (37.0) | | 0.93 (0.68-1.27) | | 0.99 (0.69-1.42) | | 60 (32.4) | | 0.76 (0.53-1.08) | | 0.75 (0.52-1.09) | |
| Issues with supply to HF | | 320 (33.5) | | 222 (42.5) | | 56 (22.8) | | **0.40*** (0.28-0.56)** | | **0.40*** (0.27-0.59)** | | 42 (22.7) | | **0.45*** (0.30-0.66)** | | **0.44*** (0.29-0.65)** | |
| Frequent stockouts at HF | | 282 (29.6) | | 188 (36.0) | | 49 (19.9) | | **0.44*** (0.31-0.64)** | | **0.47*** (0.31-0.72)** | | 45 (24.3) | | **0.57** (0.39-0.84)** | | **0.57** (0.38-0.85)** | |
| Religious/cultural beliefs | | 272 (28.5) | | 142 (27.2) | | 47 (19.1) | | **0.63* (0.44-0.92)** | | 0.75 (0.50-1.15) | | 83 (44.9) | | **2.18*** (1.54-3.09)** | | **2.46*** (1.69-3.56)** | |
| Stigma | | 207 (21.7) | | 113 (21.6) | | 56 (22.8) | | 1.07 (0.74-1.54) | | 0.97 (0.63-1.48) | | 38 (20.5) | | 0.94 (0.62-1.42) | | 0.75 (0.49-1.16) | |
| Staff shortages | | 182 (19.1) | | 144 (27.5) | | 18 (7.3) | | **0.21*** (0.12-0.35)** | | **0.26*** (0.15-0.46)** | | 20 (10.8) | | **0.32*** (0.19-0.53)** | | **0.34*** (0.21-0.57)** | |
| Staff training on SRH services | | 148 (15.5) | | 101 (19.3) | | 24 (9.8) | | **0.45** (0.28-0.73)** | | **0.49** (0.28-0.83)** | | 23 (12.4) | | **0.59* (0.36-0.97)** | | 0.61 (0.37-1.01) | |
| Patient costs | | 145 (15.2) | | 24 (4.6) | | 82 (33.3) | | **10.4*** (6.38-16.93)** | | **6.83*** (3.98-11.70)** | | 39 (21.1) | | **5.55*** (3.23-9.54)** | | **4.58*** (2.61-8.03)** | |
| No demand | | 102 (10.7) | | 40 (7.7) | | 45 (18.3) | | **2.70*** (1.71-4.27)** | | 1.30 (0.74-2.28) | | 17 (9.2) | | 1.22 (0.67-2.21) | | 1.02 (0.55-1.91) | |
| Frequent stockouts at central level | | 102 (10.7) | | 71 (13.6) | | 23 (9.4) | | 0.66 (0.40-1.08) | | 0.70 (0.39-1.25) | | 8 (4.3) | | **0.29*** (0.14-0.61)** | | **0.35** (0.16-0.75)** | |
| SRHC stockout causes | |  | |  | |  | |  | |  | |  | |  | |  | |
| Delay in supply delivery | | 471 (54.1) | | 320 (63.9) | | 83 (37.4) | | **0.34*** (0.24-0.47)** | | **0.36 (0.24-0.54)***** | | 68 (46.0) | | **0.48*** (0.33-0.70)** | | **0.52** (0.34-0.77)** | |
| What is ordered is not what HF received | | 295 (33.9) | | 226 (45.1) | | 37 (16.7) | | **0.24*** (0.16-0.36)** | | **0.31 (0.20-0.49)***** | | 32 (21.6) | | **0.34*** (0.22-0.52)** | | **0.35*** (0.22-0.54)** | |
| Problems with stock at distribution level | | 264 (30.3) | | 170 (33.9) | | 61 (27.5) | | 0.74 (0.52-1.04) | | 0.94 (0.62-1.41) | | 33 (22.3) | | **0.56** (0.36-0.86)** | | **0.61* (0.39-0.95)** | |
| Demand higher than availability | | 185 (21.2) | | 120 (24.0) | | 37 (16.7) | | **0.64* (0.42-0.96)** | | **0.49** (0.31-0.81)** | | 28 (18.9) | | 0.74 (0.47-1.17) | | 0.76 (0.47-1.23) | |
| Affordability for HF | | 138 (15.8) | | 33 (6.6) | | 67 (30.2) | | **6.13*** (3.89-9.66)** | | **5.59*** (3.27-9.53)** | | 38 (25.7) | | **4.90*** (2.94-8.16)** | | **4.82*** (2.79-8.34)** | |
| Poor stock management at HF | | 128 (14.7) | | 63 (12.6) | | 34 (15.3) | | 1.26 (0.80-1.97) | | 1.37 (0.81-2.32) | | 31 (21.0) | | **1.84* (1.14-2.96)** | | **1.84* (1.11-3.04)** | |
| Lack of storage space at HF | | 80 (9.2) | | 58 (11.6) | | 14 (6.3) | | **0.51* (0.28-0.94)** | | 0.54 (0.28-1.08) | | 8 (5.4) | | **0.44* (0.20-0.94)** | | 0.48 (0.21-1.07) | |
| Problems with medicine transport to HF | | 71 (8.2) | | 51 (10.2) | | 10 (4.5) | | **0.42* (0.21-0.84)** | | 0.49 (0.22-1.08) | | 10 (6.8) | | 0.64 (0.32-1.29) | | 0.67 (0.32-1.39) | |
| Recommendations for improvement – supply side | |  | |  | |  | |  | |  | |  | |  | |  | |
| Improve supply chain | | 523 (55.6) | | 346 (66.4) | | 104 (43.2) | | **0.38*** (0.28-0.53)** | | **0.40*** (0.27-0.57)** | | 73 (41.0) | | **0.35*** (0.25-0.50)** | | **0.38*** (0.27-0.56)** | |
| Timely supply of SRHC | | 430 (45.7) | | 274 (52.6) | | 84 (34.9) | | **0.48*** (0.35-0.66)** | | **0.48*** (0.33-0.70)** | | 72 (40.5) | | **0.61** (0.43-0.86)** | | **0.61** (0.42-0.87)** | |
| Prevent stock-outs of SRHC at HF | | 326 (34.7) | | 192 (36.9) | | 80 (33.2) | | 0.85 (0.62-1.17) | | 1.04 (0.71-1.50) | | 54 (30.3) | | 0.75 (0.52-1.08) | | 0.75 (0.51-1.10) | |
| Ensure sufficient stock available at HF | | 275 (28.7) | | 180 (34.2) | | 56 (22.6) | | **0.56** (0.40-0.80)** | | **0.65* (0.44-0.97)** | | 39 (21.2) | | **0.52** (0.35-0.77)** | | **0.57** (0.38-0.85)** | |
| Supply SRHC that were ordered | | 247 (26.3) | | 179 (34.4) | | 46 (19.1) | | **0.45*** (0.31-0.65)** | | **0.56** (0.37-0.86)** | | 22 (12.4) | | **0.27*** (0.17-0.44)** | | **0.28*** (0.17-0.46)** | |
| (Continued) staff training | | 216 (23.0) | | 140 (26.9) | | 42 (17.4) | | **0.57** (0.39-0.84)** | | **0.63* (0.41-0.97)** | | 34 (19.1) | | **0.64* (0.42-0.98)** | | 0.66 (0.43-1.03) | |
| Increase staff | | 203 (21.6) | | 143 (27.5) | | 30 (12.5) | | **0.38*** (0.24-0.58)** | | **0.51** (0.32-0.82)** | | 30 (16.9) | | **0.54** (0.35-0.83)** | | **0.57* (0.36-0.90)** | |
| Increase budget for SRHC | | 176 (18.7) | | 112 (21.5) | | 33 (13.7) | | **0.58* (0.38-0.88)** | | **0.50** (0.30-0.81)** | | 31 (17.4) | | 0.77 (0.50-1.20) | | 0.76 (0.48-1.20) | |
| Provide greater choice of SRHC | | 147 (15.6) | | 71 (13.6) | | 49 (20.3) | | **1.62* (1.08-2.42)** | | **1.60* (1.00-2.55)** | | 27 (15.2) | | 1.13 (0.70-1.83) | | 1.05 (0.63-1.73) | |
| Recommendations for improvement – demand side | |  | |  | |  | |  | |  | |  | |  | |  | |
| Client and community education | | 778 (81.1) | | 437 (82.9) | | 194 (78.2) | | 0.74 (0.51-1.08) | | 0.77 (0.50-1.20) | | 147 (79.9) | | 0.82 (0.53-1.25) | | 0.89 (0.57-1.39) | |
| Increase male partner involvement | | 357 (37.2) | | 222 (42.1) | | 82 (33.1) | | **0.68* (0.50-0.93)** | | 0.82 (0.57-1.18) | | 53 (28.8) | | **0.56** (0.39-0.80)** | | **0.57** (0.39-0.83)** | |
| Offer/improve SRH outreach services | | 280 (29.2) | | 164 (31.1) | | 62 (25.0) | | 0.74 (0.52-1.04) | | 0.77 (0.52-1.14) | | 54 (29.4) | | 0.92 (0.64-1.33) | | 0.86 (0.58-1.26) | |
| Increase choice of contraceptives | | 222 (23.2) | | 129 (24.5) | | 59 (23.8) | | 0.96 (0.68-1.37) | | 0.76 (0.50-1.16) | | 34 (18.5) | | 0.70 (0.46-1.07) | | 0.76 (0.49-1.18) | |
| Professionalise HCW-patient relationship | | 173 (18.0) | | 102 (19.4) | | 49 (19.8) | | 1.03 (0.70-1.50) | | 0.88 (0.56-1.36) | | 22 (12.0) | | **0.57* (0.34-0.93)** | | **0.43** (0.26-0.73)** | |
| Reduce costs for clients | | 202 (21.0) | | 38 (7.2) | | 113 (45.2) | | **10.68*** (7.06-16.15)** | | **7.60*** (4.79-12.04)** | | 51 (27.7) | | **5.00*** (3.13-7.88)** | | **4.10*** (2.53-6.63)** | |
| HF at times unable to provide client with SRHC and services | |  | |  | |  | |  | |  | |  | |  | |  | |
| Yes | | 359 (37.0) | | 155 (29.2) | | 123 (49.0) | | **2.33*** (1.71-3.18)** | | **1.57* (1.09-2.26)** | | 81 (42.9) | | **1.82** (1.29-2.57)** | | **1.47* (1.02-2.12)** | |
| Reasons why unable to provide client with SRHC and services | |  | |  | |  | |  | |  | |  | |  | |  | |
| SRHC was stocked out | | 131 (37.3) | | 84 (56.4) | | 35 (28.2) | | **0.31*** (0.18-0.51)** | | **0.30*** (0.16-0.56)** | | 12 (15.4) | | **0.14*** (0.07-0.28)** | | **0.11*** (0.07-0.28)** | |
| HF does not offer FP services | | 65 (18.6) | | 13 (8.8) | | 24 (19.5) | | **2.52* (1.22-5.19)** | | 1.88 (0.82-4.30) | | 28 (35.9) | | **5.82*** (2.79-12.11)** | | **6.38*** (2.97-13.72)** | |
| Client unable to pay for service | | 60 (17.2) | | 4 (2.7) | | 44 (35.8) | | **20.05*** (6.95-57.86)** | | **15.13*** (4.85-47.18)** | | 12 (15.4) | | **6.55** (2.03-21.06)** | | **6.88** (2.08-22.70)** | |
| Client was too young | | 58 (16.6) | | 19 (12.8) | | 26 (21.1) | | 1.82 (0.95-3.48) | | 1.72 (0.78-3.83) | | 13 (16.7) | | 1.36 (0.63-2.92) | | 1.15 (0.51-2.60) | |
| Service not culturally or religiously acceptable | | 56 (16.1) | | 13 (8.7) | | 5 (4.1) | | 0.44 (0.15-1.28) | | 0.42 (0.13-1.37) | | 38 (49.4) | | **10.19*** (4.94-21.01)** | | **12.65*** (5.75-27.81)** | |
| Service would not benefit client | | 25 (7.2) | | 11 (7.4) | | 9 (7.3) | | 0.98 (0.39-2.46) | | 1.26 (0.42-3.81) | | 5 (6.4) | | 0.85 (0.29-2.55) | | 0.60 (0.19-1.90) | |
| Lack of HCW knowledge | | 23 (6.6) | | 16 (10.7) | | 5 (4.0) | | **0.35* (0.12-0.98)** | | 0.53 (0.16-1.74) | | 2 (2.6) | | **0.22* (0.05-0.98)** | | **0.22* (0.05-0.99)** | |
| Client was unmarried | | 17 (4.9) | | 6 (4.1) | | 4 (3.3) | | 0.80 (0.22-2.89) | | 0.59 (0.13-2.64) | | 7 (9.0) | | 2.33 (0.76-7.20) | | 1.63 (0.49-5.45) | |
| Clients reluctant to access SRH services |  | |  | |  | |  | |  | |  | |  | |  | |  |
| Yes | 381 (39.3) | | 195 (36.7) | | 108 (43.0) | | 1.30 (0.96-1.77) | | 1.03 (0.72-1.49) | | 78 (41.5) | | 1.22 (0.87-1.72) | | 0.92 (0.64-1.31) | |  |
| Reasons for reluctance to access SRH services |  | |  | |  | |  | |  | |  | |  | |  | |  |
| Fear of stigmatisation | 238 (63.0) | | 115 (59.6) | | 70 (65.4) | | 1.28 (0.79-2.10) | | 0.69 (0.36-1.32) | | 53 (68.0) | | 1.44 (0.82-2.51) | | 0.83 (0.44-1.58) | |  |
| Patient lack of knowledge | 189 (50.0) | | 100 (51.8) | | 57 (53.3) | | 1.06 (0.66-1.70) | | 0.96 (0.53-1.73) | | 32 (41.0) | | 0.65 (0.38-1.10) | | 0.64 (0.36-1.15) | |  |
| Myths or superstitions | 169 (44.7) | | 95 (49.2) | | 43 (40.2) | | 0.69 (0.43-1.12) | | 0.86 (0.48-1.56) | | 31 (39.7) | | 0.68 (0.40-1.16) | | 0.83 (0.47-1.48) | |  |
| Religious beliefs | 148 (39.2) | | 84 (43.5) | | 33 (30.8) | | **0.58* (0.35-0.95)** | | 0.89 (0.47-1.67) | | 31 (39.7) | | 0.86 (0.50-1.46) | | 1.40 (0.76-2.59) | |  |
| Fear of side effects | 146 (38.6) | | 71 (36.8) | | 46 (43.0) | | 1.30 (0.80-2.10) | | 1.45 (0.78-2.68) | | 29 (37.2) | | 1.02 (0.59-1.75) | | 0.88 (0.48-1.62) | |  |
| Low support - male partner | 78 (20.6) | | 49 (25.4) | | 20 (18.7) | | 0.68 (0.38-1.21) | | 0.64 (0.31-1.33) | | 9 (11.5) | | **0.38* (0.18-0.83)** | | **0.43* (0.19-0.97)** | |  |
| Poverty/costs | 48 (12.7) | | 13 (6.7) | | 20 (18.7) | | **3.18** (1.51-6.97)** | | 2.14 (0.85-5.38) | | 15 (19.2) | | **3.30 (1.49-7.31)**** | | **2.46 (1.05-5.73)*** | |  |
| Frequent stock-outs at HF | 32 (8.5) | | 23 (11.9) | | 4 (3.7) | | **0.29* (0.10-0.85)** | | 0.31 (0.08-1.19) | | 5 (6.4) | | 0.51 (0.19-1.38) | | 0.58 (0.20-1.73) | |  |
| Distance to HF | 28 (7.4) | | 18 (9.3) | | 5 (4.7) | | 0.48 (0.17-1.32) | | 1.24 (0.34-4.50) | | 5 (6.4) | | 0.67 (0.24-1.86) | | 0.68 (0.21-2.15) | |  |
| Low support - female partner | 21 (5.6) | | 10 (5.2) | | 7 (6.5) | | 1.28 (0.47-3.47) | | 0.99 (0.28-3.52) | | 4 (5.1) | | 0.99 (0.30-3.25) | | 1.00 (0.27-3.72) | |  |
| Recommendations to tackle client reluctance |  | |  | |  | |  | |  | |  | |  | |  | |  |
| Expand client education | 367 (97.4) | | 189 (97.4) | | 101 (97.1) | | 0.89 (0.21-3.80) | | 0.78 (0.11-5.68) | | 77 (97.5) | | 1.02 (0.19-5.36) | | 1.39 (0.19-10.42) | |  |
| Create youth-friendly health corners | 135 (35.8) | | 76 (39.2) | | 35 (33.7) | | 0.79 (0.48-1.30) | | **0.43* (0.21-0.84)** | | 24 (30.4) | | 0.68 (0.39-1.19) | | **0.42* (0.22-0.82)** | |  |
| Involve partners | 109 (28.9) | | 67 (34.5) | | 26 (25.0) | | 0.63 (0.37-1.08) | | 0.56 (0.29-1.08) | | 16 (20.3) | | **0.48* (0.26-0.90)** | | **0.46* (0.24-0.91)** | |  |
| Staff training | 75 (19.9) | | 45 (23.2) | | 19 (18.3) | | 0.74 (0.41-1.35) | | **0.46* (0.21-0.99)** | | 11 (13.9) | | 0.54 (0.26-1.10) | | **0.43* (0.20-0.95)** | |  |
| Improve HCW-patient relationship | 63 (16.7) | | 33 (17.0) | | 17 (16.4) | | 0.95 (0.50-1.81) | | 0.84 (0.39-1.84) | | 13 (16.5) | | 0.96 (0.48-1.94) | | 0.77 (0.36-1.65) | |  |
| Improve stock availability | 57 (15.1) | | 34 (17.5) | | 15 (14.4) | | 0.79 (0.41-1.54) | | 0.56 (0.23-1.33) | | 8 (10.1) | | 0.53 (0.23-1.20) | | 0.48 (0.20-1.15) | |  |
| Empower people economically | 51 (13.5) | | 18 (9.3) | | 22 (21.2) | | **2.62** (1.33-5.16)** | | 1.70 (0.69-4.18) | | 11 (13.9) | | 1.58 (0.71-3.52) | | 1.24 (0.52-2.96) | |  |
| Reduce costs for patients | 36 (9.6) | | 5 (2.6) | | 23 (22.1) | | **10.73*** (3.94-29.22)** | | **6.97** (2.20-22.07)** | | 8 (10.1) | | **4.26* (1.35-13.45)** | | **3.47* (1.04-11.56)** | |  |
| Provide free FP services | 32 (8.5) | | 9 (4.6) | | 11 (10.6) | | 2.43 (0.97-6.07) | | 1.95 (0.66-5.77) | | 12 (15.2) | | **3.68** (1.48-9.13)** | | **3.19* (1.18-8.60)** | |  |
| CI = confidence interval; FP = family planning; HCW = healthcare worker; HF = health facility; OR = odds ratio; SRH = sexual and reproductive health; SRHC = sexual and reproductive health commodities.  ^a^The model was corrected for country, location, and level of care of the health facility.  * p<0.05, ** p<0.01, *** p<0.001 | | | | | | | | | | | | | | | | | |
