# Supplementary material for: Healthcare workers’ perspectives on access to sexual and reproductive health services in the public, private and private not-for-profit sectors: insights from Kenya, Tanzania, Uganda and Zambia
Source: BMC Health Serv Res. 2022 Jul 6;22:873. doi: 10.1186/s12913-022-08249-y (PMC9261038; doi:10.1186/s12913-022-08249-y)
Supplement: Supplementary file 3 — Additional file 3. HCWs perspectives on access to SRH barriers and recommendations for improvement, per country. Numbers represent percentage of HCWs that mentioned this barrier or recommendation. [file 12913_2022_8249_MOESM3_ESM.docx]

**Supplementary File 3. HCWs perspectives on access to SRH barriers and recommendations for improvement, per country. Numbers represent percentage of HCWs that mentioned this barrier or recommendation.**

|  | **Kenya (%)** | **Tanzania (%)** | **Uganda (%)** | **Zambia (%)** |
| --- | --- | --- | --- | --- |
| Key challenges to accessing SRHC |  |  |  |  |
| Patient lack of knowledge on SRH | 42.7 | 42.1 | 32.4 | 27.2 |
| Issues with supply to HF | 26.5 | 41.5 | 25.4 | 32.3 |
| Frequent stockouts at HF | 23.2 | 32.0 | 32.4 | 29.8 |
| Religious/cultural beliefs | 33.2 | 33.1 | 15.5 | 25.1 |
| Stigma | 33.2 | 16.1 | 22.5 | 19.6 |
| Staff shortages | 12.3 | 26.0 | 15.5 | 16.6 |
| Staff training on SRH services | 12.3 | 17.5 | 14.1 | 16.2 |
| Patient costs | 21.3 | 8.5 | 29.6 | 11.5 |
| No demand | 14.2 | 5.7 | 14.1 | 13.2 |
| Frequent stockouts at central level | 7.1 | 14.2 | 4.2 | 12.3 |
| SRHC stockout causes |  |  |  |  |
| Delay in supply delivery | 62.1 | 65.5 | 16.7 | 50.4 |
| What is ordered is not what HF received | 29.0 | 41.1 | 22.5 | 33.3 |
| Problems with stock at distribution level | 29.5 | 34.5 | 15.0 | 32.9 |
| Demand higher than availability | 12.1 | 21.9 | 25.0 | 25.9 |
| Affordability for HF | 21.1 | 12.9 | 33.3 | 6.6 |
| Poor stock management at HF | 13.7 | 15.0 | 27.5 | 8.3 |
| Lack of storage space at HF | 3.7 | 15.3 | 11.7 | 3.5 |
| Problems with medicine transport to HF | 7.4 | 8.4 | 5.8 | 9.7 |
| Recommendations for improvement – supply side |  |  |  |  |
| Improve supply chain | 51.2 | 61.1 | 28.2 | 66.8 |
| Timely supply of SRHC | 56.6 | 50.4 | 28.2 | 39.2 |
| Prevent stock-outs of SRHC at HF | 30.2 | 40.3 | 43.0 | 25.1 |
| Ensure sufficient stock available at HF | 28.1 | 35.0 | 14.8 | 27.8 |
| Supply SRHC that were ordered | 25.9 | 32.9 | 11.1 | 25.1 |
| (Continued) staff training | 25.4 | 30.1 | 14.8 | 14.5 |
| Increase staff | 15.6 | 30.1 | 14.8 | 17.5 |
| Increase budget for SRHC | 19.5 | 18.9 | 12.6 | 21.3 |
| Provide greater choice of SRHC | 23.9 | 14.8 | 9.6 | 13.2 |
| Recommendations for improvement – demand side |  |  |  |  |
| Client and community education | 82.4 | 85.3 | 76.1 | 76.8 |
| Increase male partner involvement | 34.8 | 43.2 | 30.3 | 34.4 |
| Offer/improve SRH outreach services | 37.1 | 33.6 | 25.4 | 17.8 |
| Increase choice of contraceptives | 21.0 | 22.1 | 14.1 | 32.0 |
| Professionalise HCW-patient relationship | 25.2 | 15.6 | 24.7 | 11.6 |
| Reduce costs for clients | 31.9 | 12.5 | 37.8 | 14.5 |
| HF at times unable to provide client with SRHC and services |  |  |  |  |
| Yes | 52.8 | 24.5 | 51.7 | 33.3 |
| Reasons why unable to provide client with SRHC and services |  |  |  |  |
| SRHC was stocked out | 22.7 | 57.1 | 41.9 | 47.4 |
| HF does not offer FP services | 22.7 | 19.1 | 17.6 | 13.2 |
| Client unable to pay for service | 19.1 | 6.7 | 21.6 | 22.4 |
| Client was too young | 30.9 | 11.2 | 8.1 | 10.5 |
| Service not culturally or religiously acceptable | 12.7 | 19.1 | 14.9 | 5.3 |
| Service would not benefit client | 14.6 | 5.6 | 2.7 | 2.6 |
| Lack of HCW knowledge | 2.7 | 8.8 | 9.5 | 6.6 |
| Client was unmarried | 10.0 | 0.0 | 5.4 | 2.6 |
| Clients reluctant to access SRH services |  |  |  |  |
| Yes | 54.3 | 28.6 | 55.2 | 33.1 |
| Reasons for reluctance to access SRH services |  |  |  |  |
| Fear of stigmatisation | 83.5 | 45.7 | 65.8 | 53.2 |
| Patient lack of knowledge | 52.2 | 55.2 | 46.8 | 43.0 |
| Myths or superstitions | 46.1 | 55.3 | 34.2 | 39.2 |
| Religious beliefs | 46.1 | 57.1 | 16.5 | 27.9 |
| Fear of side effects | 53.0 | 32.4 | 36.7 | 27.9 |
| Low support - male partner | 24.4 | 27.6 | 13.9 | 12.7 |
| Poverty/costs | 17.4 | 3.8 | 15.2 | 15.2 |
| Frequent stock-outs at HF | 9.6 | 11.4 | 2.5 | 8.9 |
| Distance to HF | 2.6 | 7.6 | 5.1 | 16.5 |
| Low support - female partner | 9.6 | 4.8 | 1.3 | 5.1 |
| Recommendations to tackle client reluctance |  |  |  |  |
| Expand client education | 99.1 | 97.1 | 92.5 | 100.0 |
| Create youth-friendly health corners | 60.9 | 23.8 | 22.5 | 28.6 |
| Involve partners | 36.5 | 32.4 | 17.5 | 24.7 |
| Staff training | 33.0 | 18.1 | 8.8 | 14.3 |
| Improve HCW-patient relationship | 26.1 | 14.3 | 15.0 | 7.8 |
| Improve stock availability | 20.9 | 13.3 | 10.0 | 14.3 |
| Empower people economically | 20.9 | 5.7 | 12.5 | 14.3 |
| Reduce costs for patients | 15.7 | 2.9 | 8.8 | 10.4 |
| Provide free FP services | 18.3 | 3.8 | 3.8 | 5.2 |
